# Optimizing hematite filter cake treatment using reducing agents

**DOI:** 10.1038/s41598-024-62746-0

**Published:** 2024-05-24

**Authors:** Osama Siddig, Salaheldin Elkatatny, Saad Alafnan

**Affiliations:** Department of Petroleum Engineering, King Fahd University of Petroleum and Minerals, 31261 Dhahran, Saudi Arabia

**Keywords:** Filter cake removal, Hematite, Reducing agent, Hydrochloric acid, Drilling fluid, Ferrous chloride, Chemistry, Engineering

## Abstract

In drilling operations, the formation of a filter cake is crucial for well stability, but its removal post-drilling is essential to restore rock formation productivity. This study focuses on hematite-based filter cakes and investigates factors influencing their solubility and removal, addressing a significant knowledge gap in the field. The research methodology involves examining the effects of various factors, including types and concentrations of reducing agents, temperature, particle size, and treatment duration, on the dissolution process. Notably, Nuclear Magnetic Resonance (NMR) tests are employed to assess the treatment's impact on core porosity. Among the diverse reducing agents examined, ferrous chloride emerges as the optimal choice for effectively enhancing hematite solubility. Particularly, a composite solution of ferrous chloride (10 wt.%) and hydrochloric acid (6 wt.%), was highly efficient demonstrated by exhibiting rapid solubilization of hematite filter cakes. A removal efficiency of approximately 99%, with a parallel enhancement in core permeability was achieved. NMR tests reveal the treatment's success in reinstating the porosity system, which had undergone reduction due to drilling fluid particles. Crucially, the solution exhibits a considerably lower corrosion rate than concentrated hydrochloric acid, highlighting its potential to mitigate environmental concerns while ensuring efficient filter cake removal. The findings of this research provide valuable insights into optimizing post-drilling operations, balancing environmental sustainability and operational efficiency. The identified composite solution offers a promising approach to efficient filter cake removal while mitigating environmental concerns associated with corrosion. Overall, this study contributes to advancing the understanding and practice of well productivity enhancement in the oil and gas industry.

## Introduction

Drilling fluids, commonly known as muds, play a pivotal role in enhancing drilling operations through the execution of multifarious functions. These include lubrication of subsurface tools, suspension of rock debris, and their conveyance to the surface^1,2^. Concurrently, a crucial function borne by these fluids is the maintenance of an optimal hydrostatic pressure to outbalance the pressure within formation pores. This pressure differential is important in averting unwanted inflow from the formation into the wellbore, thereby affording operational control^3^. However, this pressure overbalance precipitates the solids of drilling fluid into the penetrated formation, potentially instigating problems such as formation/ completion screens plugging, and alterations in permeability. Consequently, profound deleterious influence on well productivity/injectivity and escalation of expenditure^4–6^.

The phenomenon of filter cake (FC) formation arises when solid particulates within drilling fluids undergo precipitation at the interface of permeable rock formations, typically characterized by extremely low permeability values^7,8^. This FC formation serves to mitigate further infiltration of drilling fluid into penetrated rocks, thereby minimize potential invasion effects^9^. The attributes of the filter cake are acknowledged as pivotal properties within the domain of drilling mud studies^10–12^. Optimal filter cakes are characterized by thin profiles, impermeability, rapid generation kinetics, and facile removability^13^.

Notwithstanding its pivotal role in impeding unwanted fluid ingress during drilling, the effective removal of FC post-drilling is indispensable to facilitate efficient cementation processes and the restoration of well productivity^1,14–16^.

Filter cakes exhibit a range of distinguishing attributes encompassing porosity, thickness, permeability, and granulometric distribution. These characteristics exert a substantial influence on the extent of drilling fluid filtration^17,18^. Numerous factors impact the attributes of these filter cakes, including the nature of drilling fluid additives, salinity levels, and the composition of clay and sand within the mud^19,20^. The efficacy of filter cake treatment is influenced by various factors encompassing drilling fluid particle sizes, drilled rock porosity^21^, the formulation of drilling fluid, particularly the nature of the weighting agent, and the mud's liquid phase type^22,23^.

Empirical investigations have elucidated the preeminence of weighting materials as the principal constituent in filter cake compositions, constituting approximately 70% of the FC's mass^24^. These weighting agents are introduced to drilling muds to elevate their density, thus endowing the fluid with requisite hydrostatic pressure to surpass the pressures encountered during drilling operations. This critical mud weight increase must be achieved while concurrently upholding requisite rheological attributes and preventing the occurrence of solids sedimentation^25,26^.

Iron oxide, known as hematite, has emerged as a potential substitute for barite as a densifying additive^27^. Hematite possesses a slightly greater density relative to barite, rendering drilling fluids comparable in rheological attributes to barite-containing formulations^28^. However, hematite is beset by inherent shortcomings, notably its susceptibility to magnetism and pronounced abrasiveness, as documented in several sources^27,29,30^. Additionally, a propensity for settling exists, although this tendency can be mitigated through the micronization of hematite particles, albeit at the expense of higher fluid loss^30^.

Given that the weighting material predominantly constitutes filter cakes, the efficacy of removal solutions is largely dependent on the solubility of the weighting material within the solvent. Scholarly discourse has expounded on the treatment of drilling fluids formed with various weighting materials. However, investigations have primarily focused on commonplace agents like barite and calcium carbonate; notably, comprehensive exploration of hematite FC treatment remains wanting. The adoption of hematite as an alternative weighting agent to barite has been burgeoning, driven by escalating demands coupled with finite supplies of conventional agents.

The most effective way to dissolve hematite is by using hydrochloric acid (HCl), as the hematite is insoluble in weak acids. The reaction between hematite and HCl produces Ferric chloride (FeCl_3_) which is soluble in water.

The optimal method for hematite dissolution involves the utilization of hydrochloric acid (HCl), given hematite's insolubility in weak acids^31^. The interaction between hematite and HCl yields Ferric chloride (FeCl_3_), a water-soluble product. The hematite solubility could be enhanced by introducing reducing agent to this reaction^32^. The reducing agent, through electron donation, induces the reduction of ferric iron (Fe(III)) to ferrous iron (Fe(II)), thereby catalyze the reaction of hematite in the HCl medium.

Siddig et al.^31^, probed the dissolution of hematite filter cake using hydrochloric acid (HCl). In alignment with this trajectory, the present endeavor aims to furnish valuable insights into hematite filter cake treatment when reducing agent is incorporated within the treatment solution. The study employs an array of solubility assessments to dissect diverse influencers on the treatment process, including type and concentration of the reducing agent, temperature, particle size dispersion, and exposure period. Drawing from these foundational evaluations, a treatment is designed, followed by an experimental assessment of removal efficiency under specified conditions.

## Methods and materials

**Figure 1:**
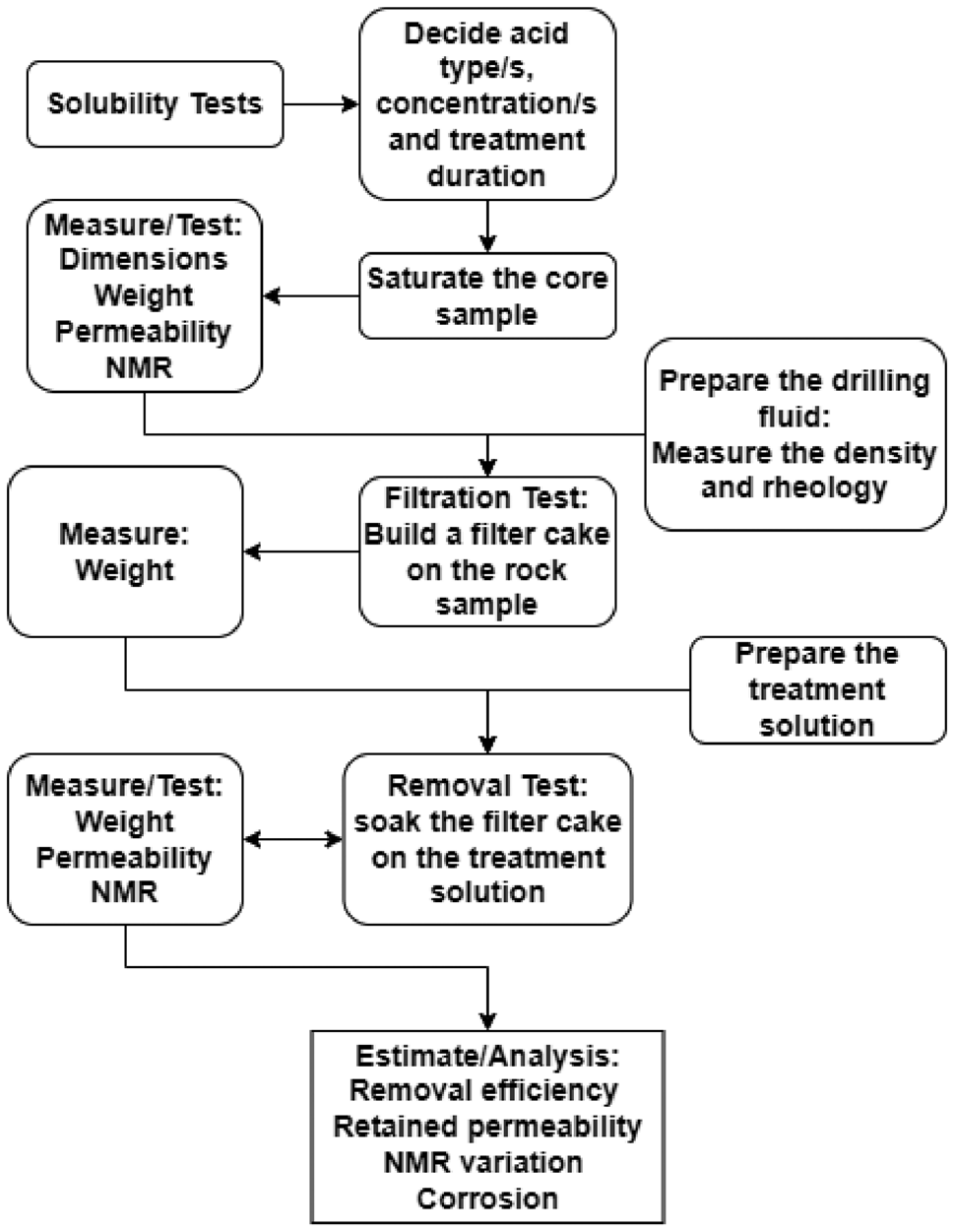
Diagrammatic representation of the research approach.

This study employs solubility assays to investigate influential factors affecting treatment efficacy. Based on the outcomes of these solubility tests, reducing agent type and concentration and treatment duration are selected and subsequently evaluated within a high-pressure high-temperature (HPHT) filtration cell. This segment elucidates the methodology of the research and the employed materials. The comprehensive methodology employed in this research is briefly summarized within a flowchart shown in Fig. 1.

### Drilling fluid

The formulation of the drilling fluid used in this study is derived from real-field operations. The corresponding formulation at the laboratory scale is detailed in Table 1. The fluid consists of an aqueous base, incorporating xanthan gum and bentonite to improve rheological properties. Filtration control is managed through the addition of starch and polyanionic cellulose polymer (PAC), with calcium carbonate (CaCO_3_) acting as a bridging agent. The fluid's alkalinity is adjusted by introducing potassium hydroxide (KOH), and potassium chloride serves as a shale inhibition agent. The drilling fluid has a density of 16.3 ppg and pH of 10.5. The rheology has been tested at atmospheric pressure and 120 °F and plastic viscosity of 24 cP, and Yield point of 35 lb/100 ft^2^.

**Table 1:** Composition of the drilling fluid.

Material	Quantity	Unit
Water	245	cm^3^
Defoamer	0.08	cm^3^
Soda ash	0.5	g
Xanthan gum	0.5	g
Starch	6	g
Bentonite	4	g
KOH	0.5	g
PAC-R	1	g
KCl	20	g
CaCO_3_ (50 microns)	5	g
Hematite	350	g

### Reducing agents

Reducing agents are critical in reduction-oxidization (redox) reactions, as they are facilitating electron transfer. The three following materials known for their reducing capabilities have been tested:

- Ferrous chloride (FeCl_2_) functions as a reducing agent owing to the ability of the Fe(II) ion to readily undergo oxidation to Fe(III). This transition involves the donation of electrons, thus facilitating redox reactions. In this study, tetrahydrate powder (Fecl_2_.4H_2_O) was utilized.
- Stannous chloride (SnCl_2_) is a versatile reducing agent characterized by the facile conversion of Sn(II) to Sn(IV), involving electron transfer. The SnCl_2_ used in this work was a dihydrate powder (SnCl_2_ · 2H_2_O).
- Oxalic acid, (COOH)_2_, exhibits reducing properties due to its ability to donate electrons in redox reactions. The molecule undergoes oxidation, producing carbon dioxide and water while facilitating the reduction of the reactant. A powder of an anhydrous oxalic acid was used in the present work.

### Solubility experiments

Solubility tests were executed to quantify the dissolution pace of hematite under diverse circumstances. A spectrum of variables was subjected to test, encompassing reducing agent concentration (upto 10 wt.%), temperature (within the range of 50–125 °C), and treatment duration (extending up to 24 h). In each trial, a mass of 4 g of the weighting material (Hematite powder) was immersed in 100 cm^3^ of the acid solution, subject to specified conditions and duration. Subsequent to testing, the residual hematite was separated through vacuum-assisted filtration, followed by desiccation. By ascertaining the post-test weight, solubility was computed utilizing Eq. (1). The solubility experimental setup is illustrated in Fig. 14 in the Appendix.

1 $$Solubility \left(wt. \%\right)=\frac{original hematite weight-remaining hematite weight}{original hematite weight}\times 100\%$$

### Filter cake removal efficiency tests

A FC was established atop a core plug via a HPHT filter press at a temperature of 100 °C and 300 psi differential pressure, maintained for a duration of thirty minutes. Subsequently, under identical parameters, the core hosting the developed filter cake underwent immersion in the removal solution for the stipulated timeframe to gauge removal efficacy. The treatment efficiency, quantified based on weight, was determined utilizing Eq. (2). The experimental setup of the filter cake removal is illustrated in Fig. 15 in the Appendix.

2 $$Removal Efficiency (wt. \%) =\frac{{\text{W}}_{2}-{\text{W}}_{3}}{{\text{W}}_{2}-{\text{W}}_{1}}\times 100\%$$

where W_1_ = untreated core weight, g, W_2_ = FC-containing core weight, g, W_3_ = treated core weight, g.

To ascertain the effectiveness of the treatment in retaining rock productivity, the permeability of the core sample underwent two distinct tests. Firstly, prior to the invasion of drilling fluid (K_1_), and subsequently, following the completion of mud filtration and filter cake removal procedures (K_2_). The retained permeability was quantified as a percentage, employing Eq. (3) for estimation.

3 $$Retained Permeability (\%) =\frac{{\text{K}}_{2}}{{\text{K}}_{1}}\times 100\%$$

Additionally, the corrosivity of the treatment solution has been estimated from the reduction on the weight of N80 steel coupon with respect to it is surface area. The corrosion test was conducted at 1000 psi and 100 °C for 6 h.

### Nuclear magnetic resonance

Nuclear Magnetic Resonance (NMR) stands as a common and reliable technique for the analysis of the porosity network within a rock sample. NMR offers a lithology-independent means of estimating porosity, enhancing the versatility and reliability of porosity estimation in various studies.

In the present study, NMR has been utilized to assess the efficiency of the treatment solution in purging particulate matter from within the rock pores. To accomplish this objective, two NMR tests were executed, initially, the examination focused on the core sample in its saturated state with distilled water, devoid of drilling fluid invasion. Subsequently, NMR analyses were performed after the treatment of the filter cake. These sequential NMR assessments served as a comprehensive means to unravel the evolving porosity dynamics and the impact of the treatment solution throughout the experimental process.

## Results and discussion

### Reducing agents

The assessment of hematite solubility in HCl encompassed three different reducing agents, namely, ferrous chloride (FeCl_2_), stannous chloride (SnCl_2_), and oxalic acid (COOH)_2_. Different concentrations of the reducing agents were added to in a 6 wt.% HCl solution to test the efficiency of each agent. The reaction between Hematite and HCl is shown in Eq. (1):

4 $${\text{Fe}}_{2}{\text{O}}_{3}+6\text{HCl}\to 2{\text{FeCl}}_{3}+3{\text{H}}_{2}\text{O}$$

Electrons are being produced from the reducing agents, as indicated by reactions 5, 6, or 7^33^, These electrons transfer to the irons in the hematite and result in partial reduction of Fe(III) to Fe(II). This redox reaction is happening simultaneously with the reactions described in Eq. (4).

5 $${\text{FeCl}}_{2}+\text{HCl}\to {\text{FeCl}}_{3}+{\text{H}}^{+}+{\text{e}}^{-}$$

6 $${\text{SnCl}}_{2}+2\text{HCl}\to {\text{SnCl}}_{4}+{2\text{H}}^{+}+{2\text{e}}^{-}$$

7 $${\text{H}}_{2}{\text{C}}_{2}{\text{O}}_{4}\to 2{\text{CO}}_{2}+{2\text{H}}^{+}+{2\text{e}}^{-}$$

**Figure 2:**
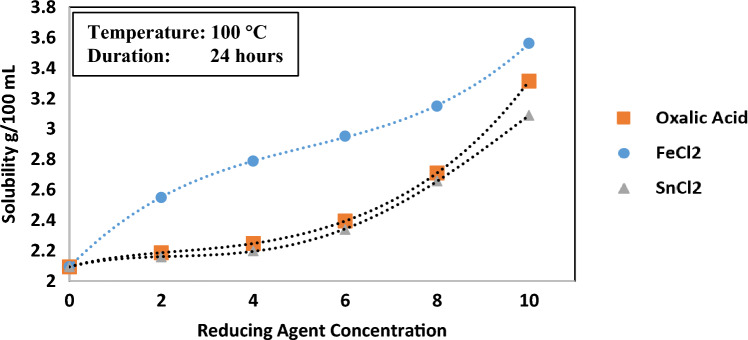
Hematite solubility with different reductants.

The investigation of hematite solubility in the acid solutions, held at 100 °C for a duration of 24 h under 120 rpm agitation speed and at atmospheric pressure, is illustrated in Fig. 2. Out of the three agents, FeCl_2_ enhanced the solubility the most, even with small concentration as 2 wt.% the hematite solubility undergo a notable increase from 2.1 g to above 2.5 g per 100 mL of the solution. The amount of the dissolved hematite reached 3.5 g at 10 wt.% concentration of FeCl_2_. No significant improvement has been noticed at low concentrations of Oxalic acid and SnCl_2_ up to 4 wt.%, however, the solubility increased at higher concentrations to reach 3.3 and 3.1 g respectively at 10 wt.% concentrations. Based on these observation, ferrous chloride will be used as a reducing agent for the further investigations.

### Agitation speed and pressure effect

**Figure 3:**
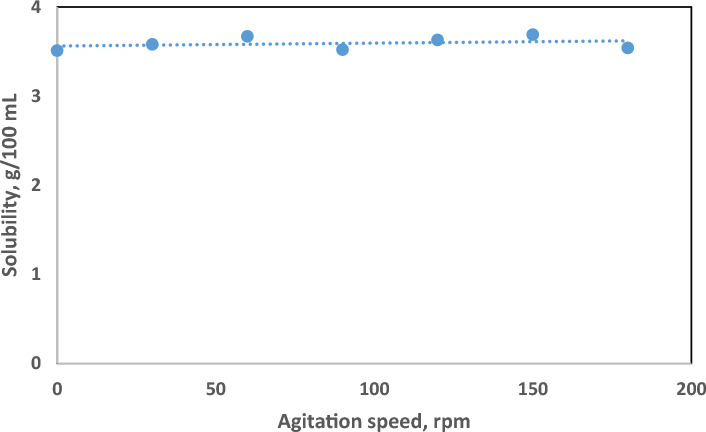
Hematite solubility under different agitation speed.

**Figure 4:**
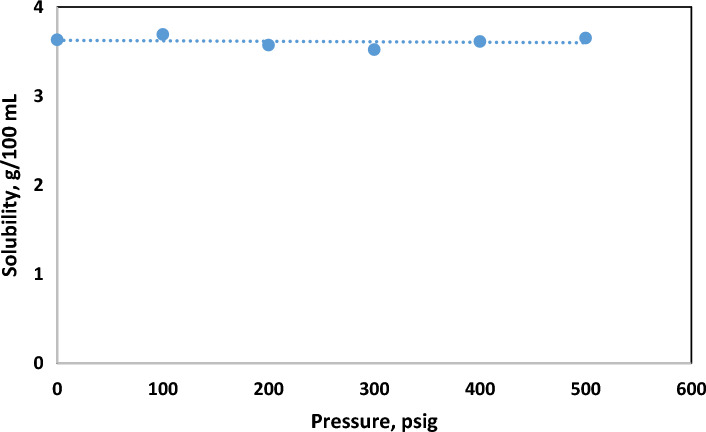
Hematite solubility at different pressure.

The influence of agitation speed and pressure on the solubility of hematite was investigated within a controlled experimental setup. Agitation speed was varied from 0 to 180 rpm, while pressure ranged from atmospheric pressure to 500 psi. The solubility tests were conducted using a treatment solution comprising 10 wt.% HCl and 10 wt.% FeCl2, with a duration of 24 h. As depicted in Fig. 3 and Fig. 4, the results indicate that within the tested range, both agitation speed and pressure exhibited negligible impact on hematite solubility.

It is noteworthy that despite variations in agitation speed and pressure, the solubility of hematite remained relatively consistent. To maintain consistency and facilitate comparison, all subsequent solubility tests in this study were conducted under uniform conditions, employing atmospheric pressure and a rotation speed of 120 rpm.

### Effect of temperature on solubility

**Figure 5:**
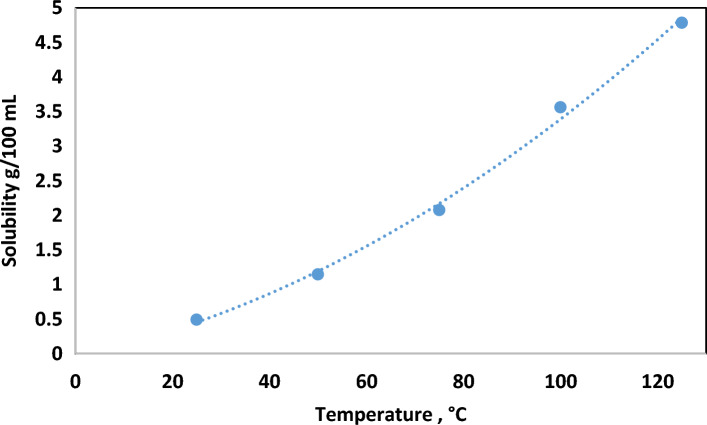
Hematite solubility in HCl solution with ferrous chloride at different temperatures.

The impact of temperature on the efficiency of hematite dissolution in the acid solution ( 6 wt.% HCl with 10 wt.% FeCl_2_) was scrutinized across a range between 25 and 125 °C for 24 h under 120 rpm agitation speed. A positive correlation between temperature elevation and solubility was discerned, exhibiting a significant increase from 0.5 g at 25 °C to 4.8 g at 125 °C. At the designated benchmark temperature of 100 °C, concurrent with other evaluations, a solubility of 3.6 g was achieved, as shown in Fig. 5.

### Particles size effect

**Figure 6:**
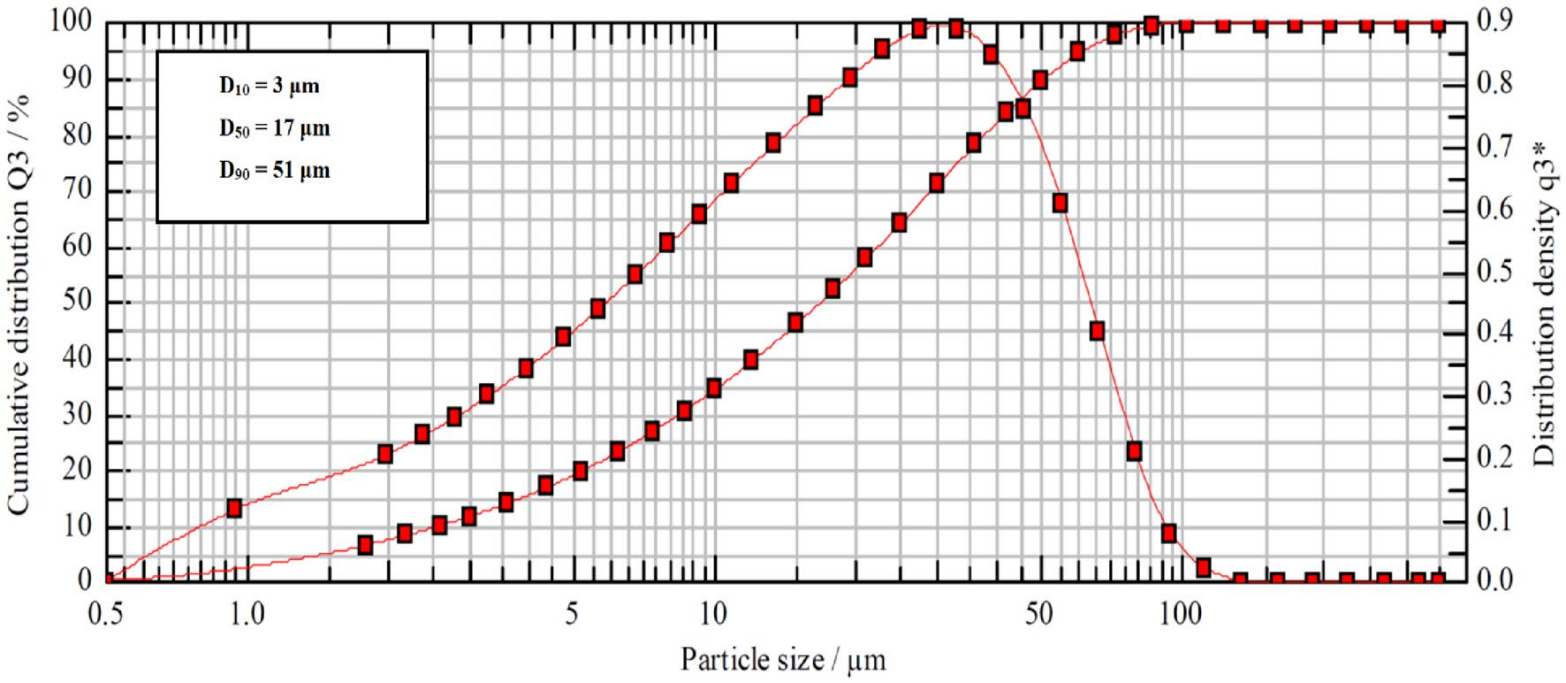
Particle size distribution of hematite.

**Figure 7:**
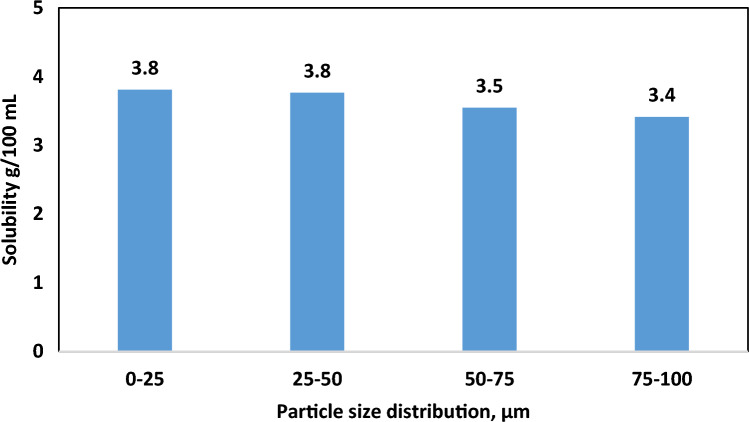
Effect of particles size on hematite solubility in the treatment solution.

Hematite particles used in this work exhibited size variations within the 5-micron to 90-micron range. Figure 6 shows the particle size distribution with a median particle size (D_50_) of 17 microns and a particle size at 90% (D_90_) of 51 microns. To discern the influence of particle size on solubility, the hematite sample was fractionated based on four sieve sizes (25 to100 microns). Subsequently, the solubility of the resultant four specimens in the acid solution (6 wt.% HCl with 10 wt.% FeCl_2_) was assessed at 100 °C for 24 h and 120 rpm agitation speed. Evidently, particle size change moderately impacts solubility, as depicted in Fig. 7. Solubility demonstrated a slight decline, ranging from approximately 3.8 g for the sub-25-micron particles to 3.4 g for particles exceeding 75 microns. This observation underscores the consequential role of particle size in hematite solubility behaviors.

### Effect of treatment duration

**Figure 8:**
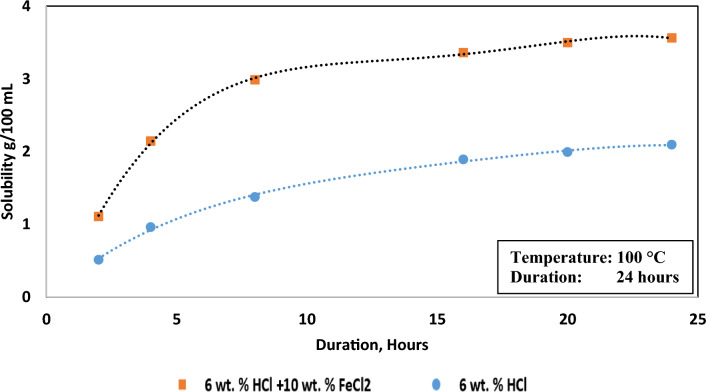
Solubility of hematite in the acid solutions over time.

The findings depicted in Figs. 2, 3, 4, 5 and 7 underwent a 24-h assessment. The hematite solubility in the HCl (6 wt.%) and the acid solution with reducing agent (6 wt.% HCl with 10 wt.% FeCl_2_) across a 24-h span, at 100 °C and 120 rpm agitation speed is illustrated in Fig. 8. In both cases, solubility exhibited an incremental trend within the initial 20 h, culminating at approximately 2.2 g. Subsequent to this period, negligible alterations ensued. The dissolution rate demonstrated a decreasing trajectory, with approximately 46% of solubility achieved within the initial 4 h in the HCl only medium and 60% when reducing agent was included. This observation indicates that the reducing agent not only augmented the solubility of hematite but also notably accelerated the dissolution rate.

### Filter cake removal efficiency

**Figure 9:**
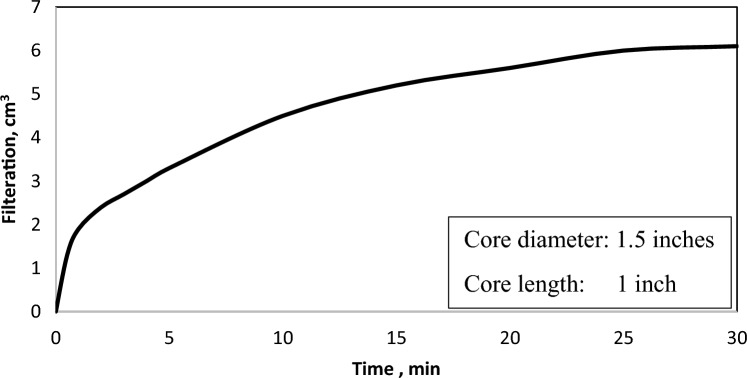
Filtration over 30 min.

The drilling fluid, incorporating hematite as a weighting material, was employed to create a filter cake on the surface of a sandstone core sample within an HPHT filtration cell. This procedure extended for 30 min under conditions of 100 °C and a 300 psi differential pressure. The total filtrate volume of 6 cm^3^, signifying the efficient performance of the filtration control additives as shown in Fig. 9. Precisely, the filtration flow stoped in 25 min, coinciding with the establishment of an impermeable filter cake formation on the surface of the core sample.

Preliminary assessments were performed to record the dimensions and weight of the core sample prior to and following the filtration process. The filter cake exhibited a slender profile with an approximate thickness of 2 mm and weighed around 4.5 g. Subsequently, the core sample containing the FC was reintroduced into the HPHT cell, replete with 200 ml of a treatment solution, and immersed in the solution for a 24-h interval at 100 °C. The treatment solution was formulated with 10 wt.% ferrous chloride and 6 wt.% hydrochloric acid.

**Figure 10:**
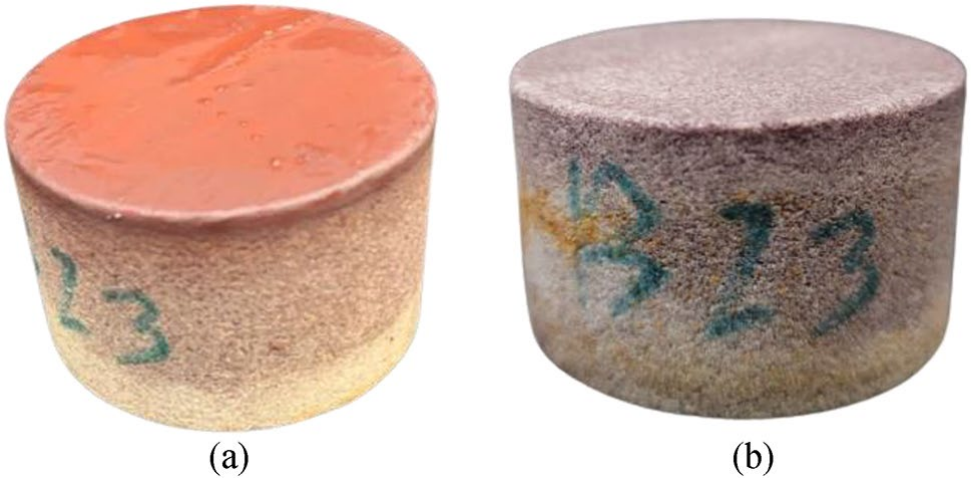
The core sample (**a**) after the formation of the filter cake and (**b**) after removal.

Upon completion of the treatment, the core sample was retrieved, measured, and the treatment efficiency was quantified using Eq. (2). The resulting tratment efficiency was around 99%, is visually corroborated in Fig. 10, demonstrating the successful elimination of the filter cake from the core's surface. Additionally, the core permeability was measured before the filteration and after the treatment and the retained permeability was estimated using Eq. (3) to be around 98% of its original value as shown in Table 2.

**Table 2:** Filter cake removal test results.

Before the filteration test test	
Core porosity	18.2%
Core permeability	351.2 mD
Core weight	69.36 g
After filteration	
Core weight with filter cake	73.84 g
Filter cake thickness	2 mm
After removal	
Core weight	69.41 g
Core permeability	345.3 mD
Treatment effeciancy	
Removal effecincy	98.8%
Regained permeability	98.3%

### Nuclear magnetic resonance

**Figure 11:**
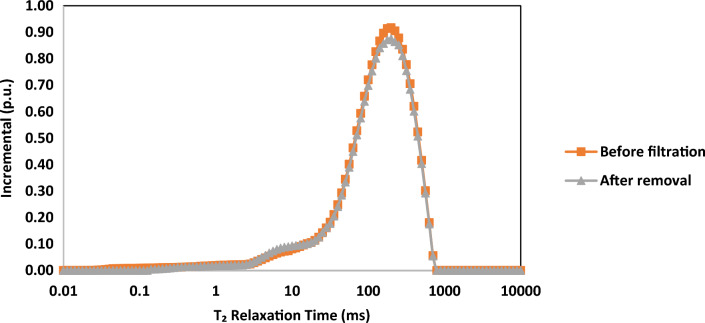
Incremental porosity against T_2_ relation time for core sample at three stages.

**Figure 12:**
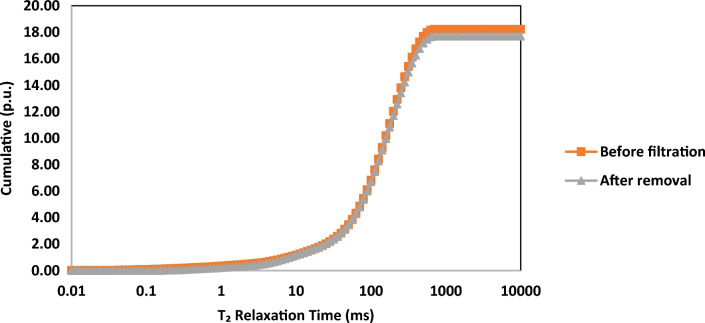
Cumulative porosity against T_2_ relation time for core sample at two stages.

The NMR analysis was carried out in two distinct stages of the core sample. Initially, the investigation was centered on the core sample saturated with distilled water, prior to any introduction of drilling fluid. Subsequent assessments were performed after the comprehensive treatment of the filter cake. Figures 11 and 12, respectively, depict the incremental and cumulative porosity distributions as functions of t_2_ relaxation time. The smaller t_2_ value is indicative of more confined pore sizes, while conversely, larger values suggest the presence of relatively larger pores.

In Figs. 11 and 12, the similarity between the two curves suggests that the removal treatment effectively restores the porosity system. This treatment not only eliminates the filter cake but also clears any precipitates within the core pores, closely resembling the initial state of the formation. According to this observation, the NMR results cope with the treatment efficiency estimated in the previous section.

### Corrosion test

The evaluation of the treatment solution's corrosive potential was undertaken through its exposure to N80 steel coupons under controlled conditions, involving a temperature of 100 °C and a pressure of 2500 psi, for a duration of 6 h. The corrosion kinetics of this solution were contrasted against three benchmarks, namely, 10 wt.% ferrous chloride in isolation, 6% HCl and a 12% concentrated HCl solution, which is the concentration needed to achieve higher hematite solubility.

**Figure 13:**
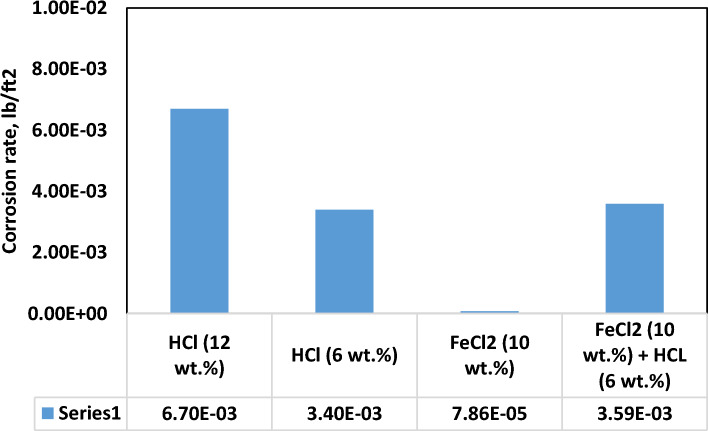
Corrosion of steel coupon in HCl, ferrous chloride and mixture.

**Figure 14:**
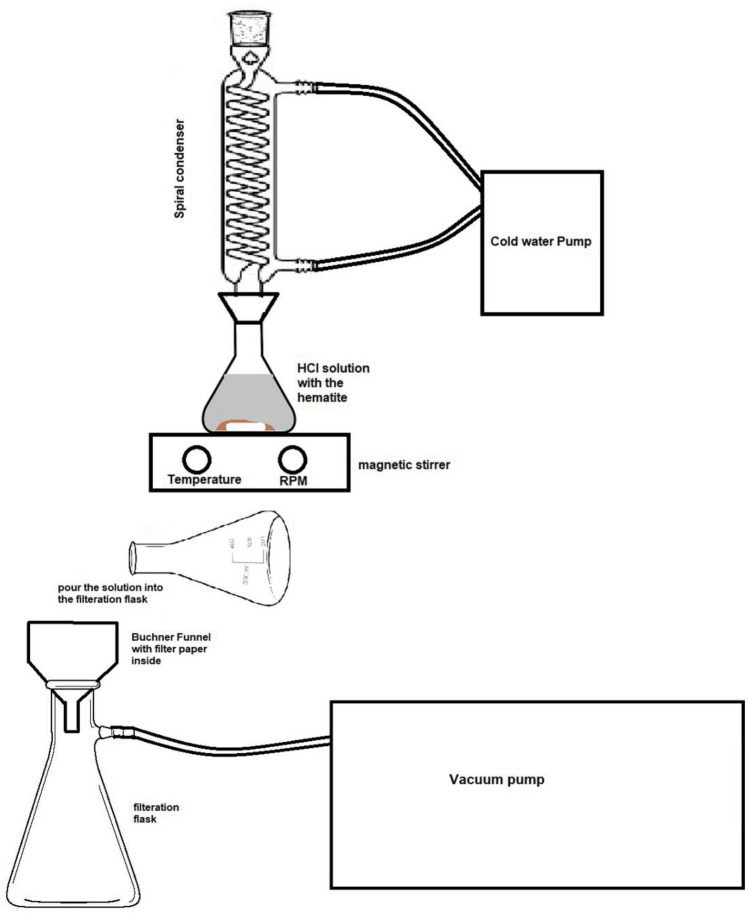
Solubility test setup.

**Figure 15:**
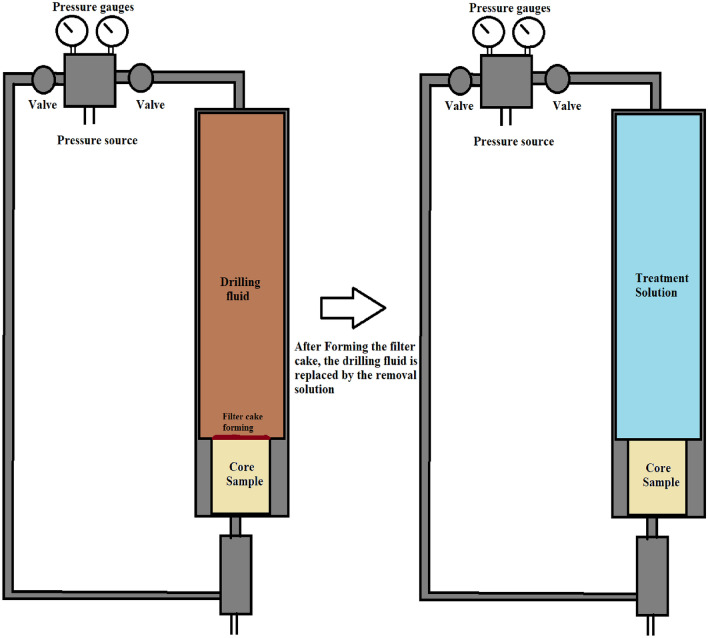
Schematic of the filter cake removal test.

Remarkably, the corrosion rate, quantified at 0.00359 lb/ft^2^, was found to be notably lower in comparison to the corrosion induced by the concentrated HCl solution, which registered a corrosion rate of 0.0067 lb/ft^2^ under identical experimental parameters. This corrosion rate is nearly identical to the corrosion of 6% HCl solution, as evident in Fig. 13.

## Conclusions

The treatment of filter cake formed by a drilling fluid containing hematite using HCl acid solution with reducing agent has been investigated in this study. The research encompassed the evaluation of various factors influencing treatment efficiency, including reducing agent type and concentration, particle size, temperature, and duration of the treatment. The key findings summarized herein are as follows:

- Among the reducing agent tested, ferrous chloride exhibited higher improvement of hematite solubility. The presence of the reducing agent not only increased the solubility of hematite but also notably accelerated the dissolution rate.
- Temperature was found to exert a more pronounced impact on treatment efficiency compared to particle size distribution.
- The utilization of a 6 wt.% HCl acid solution with 10 wt.% ferrous chloride as reducing agent effectively removed a filter cake adhering to a sandstone core sample, achieving a removal rate around 99%, with a similar effect on restoring permeability.
- The application of NMR analysis at various stages of the study affirmed that the treatment was efficacious in restoring the porosity system, which had been altered by drilling fluid particles.
- The tested corrosiveness of the treatment solution was markedly lower when contrasted with HCl concentrations that exhibit hematite solubility within a similar range. This underscores the suitability of the treatment solution in minimizing corrosion concerns during treatment operations.

## Data Availability

No external data was used for this research. All the generated experimental data are included in this manuscript. The datasets used and/or analysed during the current study available from the corresponding author on reasonable request.

## Appendix

See Figs. 14,15.
